# Review of microbial touchscreen contamination for the determination of reasonable ultraviolet disinfection doses

**DOI:** 10.3205/dgkh000401

**Published:** 2021-11-02

**Authors:** Martin Hessling, Robin Haag, Ben Sicks

**Affiliations:** 1 Ulm University of Applied Sciences, Institute of Medical Engineering and Mechatronics, Ulm, Germany

**Keywords:** touchscreen, contamination, staphylococci, ESKAPE pathogens, disinfection, ultraviolet radiation, UVC, UVB, UVA, far-UVC

## Abstract

**Background:** Touchscreens are usually microbially contaminated and can therefore act as fomites inside and outside healthcare environments. Due to the increasing use of such touchscreens and the growing awareness of infection risks, approaches that allow safe and automatic disinfection are desired. Ultraviolet (UV) irradiation, with its known antimicrobial efficacy, could achieve this goal, but should be executed with limited touchscreen degradation, disinfection duration, and energy consumption. It should also pose as little harm as possible to humans even in case of failure.

**Materials and methods:** A literature search was performed first to identify the microorganisms most commonly found on touchscreens. Then, the 90% reduction doses (D90 doses) for the different relevant microorganisms and UV spectral ranges were determined from the literature, and irradiation doses are suggested that should reduce most of these important microorganisms by 5 log-levels.

**Results:** The most frequent microorganisms are staphylococci, bacilli, micrococci, enterococci, pseudomonads and *E. coli* with small differences between hospital and community environments, if antibiotic resistance properties are ignored. The determined irradiation doses for a 5 log-reduction of the most frequent microorganisms are about 40 mJ/cm^2^, 80 J/cm^2^, 500 J/cm^2^ and 50 mJ/cm^2^ for the UV spectral ranges UVC, UVB, UVA and far-UVC, respectively. These doses are also sufficient to inactivate all nosocomial ESKAPE pathogens on touchscreens by at least 99.999%.

**Conclusion:** Disinfection is achievable in all UV spectral ranges, with UVC being the most effective, enabling automatic disinfection within a minute or less. The much higher doses required in the UVB and UVA spectral range result in much longer disinfection durations, with the advantage of a reduced risk to humans. For all kinds of UV irradiation, the doses should be limited to reasonable values to avoid irradiating an already more or less sterile surface and to prevent degradation of touchscreen devices.

## Zusammenfassung

**Hintergrund:** Touchscreens weisen meist mikrobielle Kontaminationen auf, die innerhalb und außerhalb von Gesundheitseinrichtungen zu Infektionen führen können. Aufgrund des zunehmenden Einsatzes von Touchscreens und des wachsenden Hygiene-Bewusstseins werden Ansätze gesucht, die eine sichere und möglichst automatische Desinfektion ermöglichen. Ultraviolette (UV) Bestrahlung mit ihrer bekannten antimikrobiellen Wirkung könnte dieses Desinfektionsziel erreichen, doch sollte dies mit einer sinnvollen Begrenzung der Touchscreen-Degradation, der Desinfektionsdauer und des Energieverbrauchs erfolgen und auch im Fehlerfall Menschen möglichst wenig schädigen.

**Material und Methoden**: Zunächst wird eine Literaturrecherche durchgeführt, um die am häufigsten auf Touchscreens vorkommenden Mikroorganismen zu identifizieren. Dann werden die 90%- Reduktionsdosen (D90-Dosen) für die verschiedenen Mikroorganismen und UV-Spektralbereiche aus der Literatur ermittelt und Bestrahlungsdosen vorgeschlagen, die die meisten der relevanten Mikroorganismen um 5 Log-Stufen reduzieren.

**Ergebnisse:** Die am häufigsten gefundenen Mikroorganismen sind Staphylokokken, Bazillen, Mikrokokken, Enterokokken, Pseudomonaden und *E. coli* mit geringen Unterschieden zwischen Gesundheitseinrichtungen und nicht-medizinischen Umgebungen, wenn Antibiotikaresistenzen nicht betrachtet werden. Die ermittelten Bestrahlungsdosen für eine 5 Log-Reduktion der häufigsten Mikroorganismen liegen bei etwa 40 mJ/cm^2^, 80 J/cm^2^, 500 J/cm^2^ und 50 mJ/cm^2^ für die UV-Spektralbereiche UVC, UVB, UVA bzw. Far-UVC. Diese Dosen reichen auch aus, um alle nosokomialen ESKAPE-Erreger auf Touchscreens um mindestens 99,999% zu inaktivieren.

**Schlussfolgerung:** Eine Desinfektion ist in allen UV-Spektralbereichen möglich, wobei UVC am wirksamsten ist und eine automatische Desinfektion innerhalb einer Minute oder weniger ermöglicht. Die viel höheren benötigten Dosen im UVB- und UVA-Spektralbereich führen zu einer deutlich längeren Desinfektionsdauer mit dem Vorteil eines geringeren Risikos für den Menschen. Bei allen Arten der UV-Bestrahlung sollten die Dosen auf vernünftige Werte begrenzt werden, um die Bestrahlung einer bereits mehr oder weniger sterilen Oberfläche zu vermeiden und um die Degradation von Touchscreen-Geräten zu minimieren.

## Introduction

The corona pandemic has raised the awareness for hygiene, microbial contaminations and the importance of disinfection. However, it is not always easy to disinfect all objects and surfaces that are potentially contaminated. This is especially true with the increasing use of touchscreens in all areas of life. While 10 years ago only smartphones and tablet computers bore touchscreens, which were often used by a single person, there are now more or less public touchscreens, e.g., on photocopiers or coffee machines in companies and public ticket machines or ATMs that can be accessed by a large number of people in a short time.

Due to the necessary direct contact of human fingers with the touchscreen, the release and ingestion of microorganisms (including pathogens) is virtually unavoidable. Therefore, it is not surprising that various studies find microbial contamination on up to 100% of touchscreens and that there is concern that such screens are potential vectors of infection [1], [2], [3], [4], [5], [6], [7], [8]. Consequently, touchscreen disinfection is recommended or even demanded [4], [9], [10], [11]. 

Chemical disinfectants such as ethanol, hypochlorite and chlorhexidine, as well as ultraviolet irradiation in the spectral range of 200–280 nm, are known to offer good antimicrobial properties [12], [13], [14], [15], [16], [17], [18], [19], [20], [21]. Unfortunately, both techniques also suffer some significant drawbacks. 

The application of chemical disinfectants is prohibited by some device manufacturers and typically requires manual execution, limiting potential applications [22], [23]. Muniz de Oliveira et al. fear that typical hospital disinfectants might damage devices like tablets and smartphones [21], although ethanol-impregnated wipes, for instance, are allowed at least for some touchscreen devices [24], [25]. They are successful against many bacteria [12], [13], [26], but not against *Clostridioides difficile* for instance and some viruses [12].

Ultraviolet radiation is divided into different ranges depending on the wavelength: UVA: 400–315 nm, UVB: 315–280 nm and UVC: 200–280 nm. UVC exhibits the strongest antimicrobial effect by damaging the DNA and RNA of all microorganisms without exception. Disinfection by ultraviolet radiation is a process that can be performed in minutes or even seconds given sufficient irradiance [8], [14], [15], [16], [17], [18], [19], [20], [27], [28]. Conceivable applications are those in which this could happen automatically even between two users, e.g., by irradiating the touchscreen from above or from the side, or scanning it as described by Alhmidi et al. [27]. 

Unfortunately, UVC does not only affect microorganisms, but can also be harmful to humans. However, within UVC. there is the spectral range of 200–230 nm, called far-UVC, which is assumed to be similarly effective as the 254 nm UVC radiation of the widely applied mercury vapor lamps, but poses a much lower risk to humans [29], [30]. Unfortunately, suitable radiation sources are still difficult to obtain and many properties of far-UVC have not yet been sufficiently investigated. UVA and UVB radiation also exhibit antimicrobial effects, but require much higher irradiation doses and thus generally longer irradiation durations compared to UVC. Nevertheless, UVA and UVB can also harm people and damage materials.

In a recent investigation by Khazova et al. [31], 48 commercial home-use UV disinfection devices were investigated for their coronavirus reduction potential and safety to skin and eye. Most of them contained UVC emitters, but also some UVA emitters or even visible light sources. Most of these commercial devices did not exhibit convincing disinfection properties, but posed a threat to humans. 

Apart from the possible risk to humans, there are other reasons to limit the irradiation dose for touchscreens to a reasonable level. Touchscreens are technical devices in which, for example, plastics, adhesives or organic light-emitting diodes (OLEDs) are used. All these materials can degrade under UV irradiation and change their physical properties for the worse [32], [33], [34], [35], [36], [37], [38], [39], [40]. Lastly, it should be mentioned that shorter irradiation durations are usually achieved faster and are more environmentally friendly, as less energy and fewer UVC lamps with toxic mercury are required.

Therefore, in the study presented here, the most relevant microorganisms on touchscreens were identified on the basis of scientific publications. With the help of published UV disinfection results, touchscreen irradiation doses for UVC, UVB, and UVA and Far-UVC were determined which result in a 5 log-reduction for the majority of the most frequent touchscreen microorganisms. The question of how often a touchscreen should be reasonably disinfected, especially in a community environment, was not addressed.

## Materials and methods

The first step was to identify the most common microbial contaminants on touchscreens. With various combinations of the terms “touchscreen”, “touch display”, “information kiosk”, “electronic menu”, “e-tablet”, “tablet”, “teller machine”, “banking machine”, “computer kiosk”, “contamination”, “bacteria”, “pathogens”, “fungi”, “viruses”, “microorganisms”, “disinfection”, “reduction”, “inactivation”, and “photoinactivation”, a literature search was performed in Pubmed and Google Scholar. References in the retrieved literature were examined for their possible inclusion in this study, as were references citing the identified literature. 

Cellphone studies were only included if the phones predominantly had touchscreens. In studies in which touchscreens and key pads were analyzed separately, only the touchscreen data was retrieved. To increase the clarity of this study, among the microorganisms and pathogens found in the individual studies, only those detected in at least 5% of the analyzed samples were considered.

The next step was to count how often the different microorganisms were mentioned in the individual studies. Only the most common ones, whose genera were mentioned at least 5 times (in about 10% of all studies), were included in the further analysis. It was noted whether the contamination studies were performed in a healthcare-related environment. This included touchscreens of healthcare workers, laboratory workers in hospitals, medical students and hospital inpatients. All these results were marked as “hospital” and all others as “community” studies.

For the most frequently mentioned microorganisms, UV irradiation doses for a 90% reduction (D90 dose) were searched in the literature. When multiple results were available, the median of the D90 doses for the genus was determined. This was performed separately for the spectral ranges UVC, UVB, UVA and far-UVC. Afterwards, for each of these ranges, a UV dose was determined which would lead to a 5-log reduction (99.999%) of the majority of the most important microorganisms. 

## Results

About 46 “hospital” and 24 “community” studies were retrieved that met the above mentioned criteria (see Attachment 1). Most of these involved touchscreens of cell phones in a medical environment. Public touchscreens, such as those of cash dispensers, have been relatively rarely investigated. 

Touchscreen contamination rates of 90% and higher were found in about half of the studies, and staphylococci were identified in virtually all of them. Neither of these findings is surprising, given that staphylococci commonly colonize skin and touchscreens are handled with fingers. 

A list of the other most frequently mentioned microorganisms can be found in Table 1 (Tab. 1), which specifies all those whose genera were detected at least 5 times in the individual studies. The compilation of this table was somewhat complicated by non-specific determinations such as “gram-negative rods”, “diphtheroid”, “coliforms”, “enterobacteriaceae”, “yeast” and similar descriptions. 

The frequency of detected microorganisms on touchscreens in descending order is *Staphylococcus spp.*, *Bacillus spp*., *Micrococcus spp*., *Pseudomonas spp*., *E. coli*, *Enterococcus spp*., *Klebsiella spp*., *Streptococcus spp*., *Corynebacterium spp*., and *Acinetobacter spp*. 

In some studies, the authors quantitatively reported contamination as colony forming units (CFU) per square centimeter or per device [11], [28], [41], [42], [43], [44], [45], [46], [47], [48], [49], [50], [51], [52]. When these data are combined for analysis, assuming a smartphone touchscreen with an area of 100 cm^2^, a very inconsistent picture emerges. Typical total contamination levels are in the range of 1,000 CFU per device, but there are large outliers above and below this value, with a maximum of 60,000 CFU reported on a single public touchscreen [48].

For the microorganisms in Table 1 (Tab. 1), D90 doses for different UV spectral ranges were searched in the literature and presented in Table 2 (Tab. 2), together with suggestions for a reasonable irradiation dose. Surprisingly, although UV disinfection has been known for more than a hundred years, there is no published data for some combinations of relevant microorganisms and UV spectral ranges.

Each suggested irradiation dose was chosen to reduce all or as much as reasonably possible of the relevant microorganisms by 5 log-levels, which is facilitated by the fact that the most important microorganisms – staphylococci, bacilli, micrococci, pseudomonads, *E. coli* and enterococci – are quite UV sensitive. Taking roughly 5 times the D90 dose of the known least UV-sensitive microorganism should work for UVC, UVB and UVA. For far-UVC, the streptococci were ignored, because the high D90 value is based on a single study and streptococci are not among the most important microorganisms in this investigation. Additionally, the suggested dose is still sufficient for a 2.5 log-reduction of *Streptococcus spp*.

## Discussion and conclusion

The relevant microorganisms identified here are largely consistent with previous studies [7], [53], [54], [55]. Among gram-positive bacteria, staphylococci, bacilli and micrococci are the most prevalent, and among the gram-negative bacteria, the most common are pseudomonads and *E. coli*. It is worth mentioning that in this analysis – which did not consider antibiotic resistances – there is no significant difference between the hospital and the community studies. However, there are two exceptions: *E. coli* is the second-most frequent microorganism in the community studies and Acinetobacter spp. do not play a significant role on community touchscreens. It is also worth noting that the most notorious nosocomial pathogens, the so-called ESKAPE pathogens *Enterococcus faecium*, *Staphylococcus aureus*, *Klebsiella pneumoniae*, *Acinetobacter baumannii*, *Pseudomonas aeruginosa*, and *E. coli*/ *Enterobacter spp*. [56], are among the microorganisms listed in Table 1 (Tab. 1).

So far, no infective coronaviruses have been found on touchscreens, but in the light of the ongoing coronavirus pandemic, it may be surprising that only a few viruses and fungi have been observed at all. This does not necessarily mean that touchscreens are mostly free of viruses and fungi, but might be at least partly due to the fact that only a few of the individual touchscreen studies explicitly looked for viruses (5) and fungi (13). In almost all of these virus and fungi studies, viruses (or virus RNA) and fungi were actually found, so it can be assumed that a larger number of corresponding studies might also have detected more viruses and fungi.

The suggested irradiation dose should lead to at least a 5 log-reduction of the most frequent microorganisms. It should be mentioned that a 99.999% reduction is not only a widespread disinfection goal but also a very reasonable one in this application. Even the most highly contaminated device with 60,000 CFU [48] would have less than 1 surviving bacterium after applying the suggested irradiation. Higher doses would result in an unnecessary irradiation of an already more or less sterile touchscreen, consume time and energy, and accelerate degradation. It should be reiterated that all suggested UV irradiation doses are also effective against the notorious ESKAPE pathogens in the hospital/healthcare environment.

The suggested UVC irradiation dose of about 40 mJ/cm^2^ in Table 2 (Tab. 2) is in accordance with the international standard for drinking water disinfection, EN 14897, which requires at least 40 mJ/cm^2^ [57]. This UVC dose also roughly matches the 60 mJ/cm^2^ applied by Liebermann et al. [14]. 

The necessary or suggested far-UVC irradiation dose of approx. 50 mJ/cm^2^ is somewhat higher but in a range similar to that of the UVC dose, although far-UVC-D90 data does not yet exist for some microorganisms, including the relevant micrococci. However, this ratio between UVC and far-UVC doses for the selected microorganisms is also in agreement with our previous study, which included a larger number of microorganisms [30]. 

It should be noted that all UVC and far-UVC applications might fail in the presence of touchscreen scratches. The top glass layer is assumed to exhibit low transmission in the complete UVC region, and therefore microorganisms deep in a scratch are at least partially shielded against irradiation. 

This undesirable effect due to scratching should be much less pronounced for UVB and UVA radiation, but here, irradiation doses of approx. 80 and 500 J/cm^2^ are necessary, which are higher than the UVC suggestions by a factor of 2,000 and 10,000, respectively. Since the power and irradiance of UVB and UVA cannot be arbitrarily increased, such doses are only possible with irradiation durations that are far above possible UVC disinfection times. Therefore, rapid disinfection in the range of minutes hardly seems technically feasible with UVB and UVA radiation, but there remains – especially for the UVA range – the advantage of a reduced hazard to humans.

Finally, we would like to emphasize that this investigation is about reasonable UV-disinfection doses in a hospital and community environment. The necessary frequencies of such disinfection procedures are probably much lower in a community setting than in a hospital, but a recommendation for UV irradiation frequencies was not determined in this study.

## Notes

### Competing interests

M. Hessling has filed a patent application on surface disinfection. B. Sicks and R. Haag have nothing to declare.

### Acknowledgment

This work was financially supported by the German Federal Ministry of Economics and Technology as part of the ZIM joint project “Clean Screen” (grant number KK5191602LU1).

## Supplementary Material

Detected microbial contaminations on touchscreens

## Figures and Tables

**Table 1:**
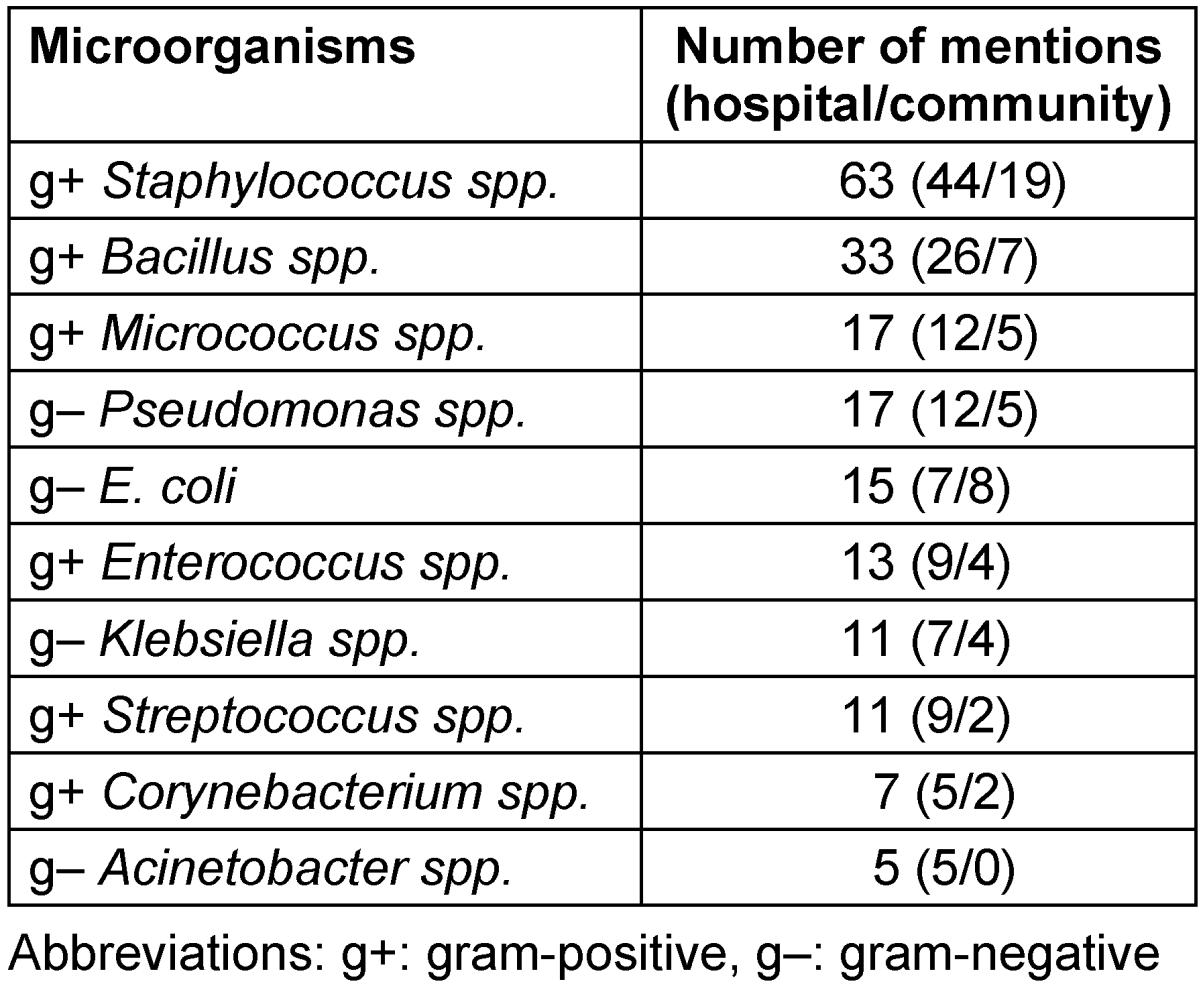
List of microorganisms (genera) found most often and at least 5 times

**Table 2:**
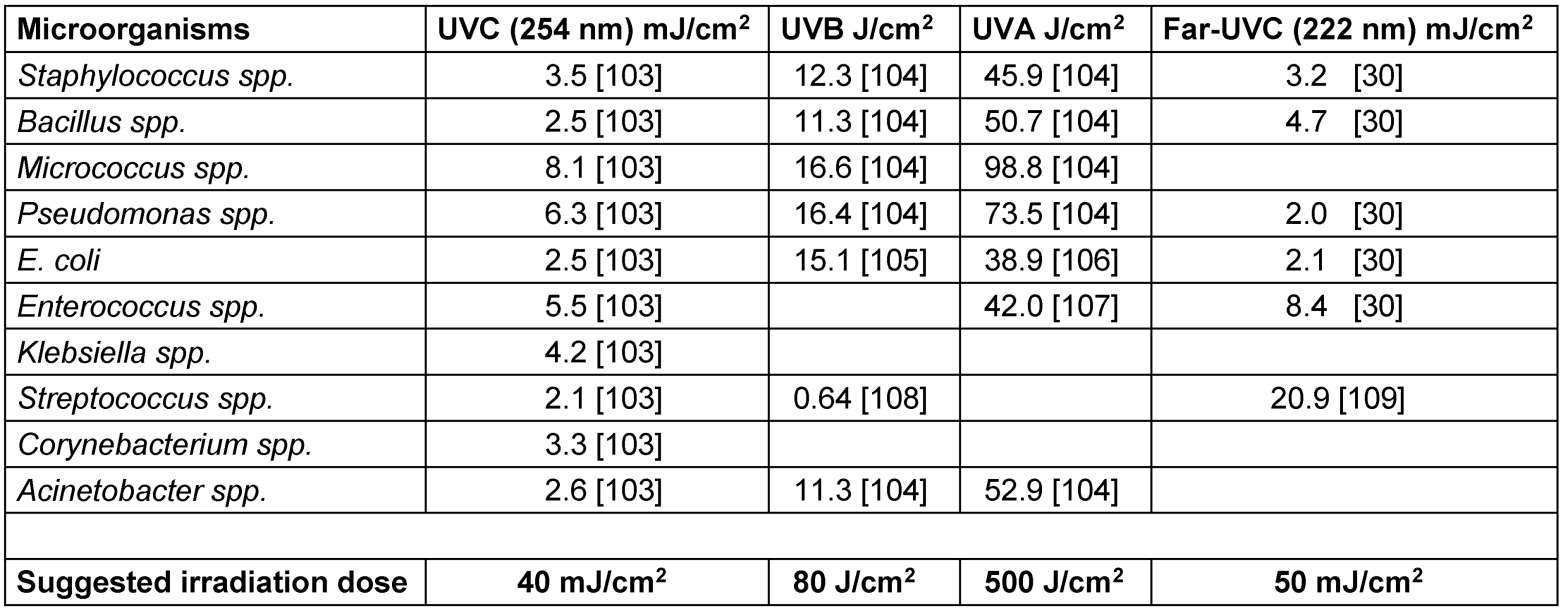
Relevant microorganisms/genera and published D90 doses for different UV ranges in different units supplemented by suggestions for a reasonable irradiation dose
