# Supplementary material for: Review of microbial touchscreen contamination for the determination of reasonable ultraviolet disinfection doses
Source: GMS Hyg Infect Control. 2021 Nov 2;16:Doc30. doi: 10.3205/dgkh000401 (PMC8662742; doi:10.3205/dgkh000401)
Supplement: Detected microbial contaminations on touchscreens [file HIC-16-30-s-001.pdf]

Attachment 1

Detected microbial contaminations on touchscreens

Hospital	Community	Touchscreen device	Reference
g+ *Staphylococcus spp.*<br>CNS 11.1%<br>*S. aureus* 15.6%	g+ *Staphylococcus spp.*<br>CNS 31%<br>*S. aureus 20%*<br>*Enterococcus faecalis* 5.2%<br>g– *E. coli* 6.5%	Mobile phones	[58]
g+ *Staphylococcus spp.*<br>CNS 76.5%<br>*S. aureus* 6.9%		Mobile phones	[59]
g+ *Staphylococcus spp*. 65%<br>*S. warneri* 40%<br>*S. epidermis* 30%<br>*S. capitis* 10%<br>*S. sciuri* 10%<br>*S. xylosus* 5%<br>*S. aureus* 5%<br>g– *enterobacteriaceae* 39%		Mobile phones	[41]
	g+ *Staphylococcus spp.*<br>CNS 96%<br>*S. aureus* 8%<br>*Bacillus spp.* 36%<br>*Micrococcus spp.* 54%<br>g– diphtheroids 20%	Mobile phones	[9]
g+ MRSA 15%		Tablets	[60]
g+ *Staphylococcus spp.*<br>*S. epidermis* 26.2%<br>*S. hominis* 18.4%<br>*S. capitis* 14.3%<br>other CNS 5.3%<br>*Micrococcus luteus* 22.2%		Tablets	[42]
g+ MRSA (Staphylococcus) and<br>VRE (Enterococcus) 13%		Mobile phones	[43]
g+ *Staphylococcus spp.*<br>CNS 96.9%<br>*S. aureus* 73.2%<br>MRSA 50.9%<br>*Enterococcus spp*. 100%<br>g– *P. aeruginosa* 6.7%		Tablets	[3]
g+ *Staphylococcus spp*.<br>CNS 42.3%<br>*S. aureus* 63.1%<br>*Enterococcus faecalis* 14.4%		Mobile phones	[61]
	g+ *Staphylococcus spp*. 23%<br>*Streptococcus spp*. 26.4%	Mobile phones	[62]
g+ *Staphylococcus spp*.<br>CNS 47.4%<br>*Micrococcus spp*. 5.4%<br>*Streptococcus viridans* 5.4%		Mobile phones	[63]
	g+ *Staphylococcus spp*. 25%<br>*S. capitis* 10%<br>*S. epidermis* 10%<br>*Micrococcus spp*. 20%<br>*Micrococcus luteus* 35%<br>g– *Moraxella spp.* 15%	Mobile phones	[44]

Hospital	Community	Touchscreen device	Reference
g+ *Staphylococcus spp.*<br>CNS 60.6%<br>*S. aureus* 25.8%<br>*Bacillus spp.* 13.6%	g+ *Staphylococcus spp.*<br>CNS 31.8%<br>*S. aureus* 24.2%	Mobile phones	[45]
	g+ *Staphylococcus spp*. 96.5%<br>*S. aureus* 81.5%<br>MRSA 6.5%	Mobile phones	[64]
g+ *Staphylococcus spp*.<br>CNS 86.7%<br>*S. aureus* 50%<br>*Bacillus spp*. 13.3%<br>*Micrococcus spp*. 7.8%<br>*Enterococcus spp*. 15.6%<br>*Streptococcus spp*. 12.2%<br>g– *Acinetobacter bauman*. 5.6%<br>diphtheroids 10%<br>f yeasts 6.7%<br>f molds 20%	g+ *Staphylococcus spp.*<br>CNS 76.1%<br>*S. aureus* 56%<br>*Bacillus spp*. 11.9%<br>*Micrococcus spp*. 15.%<br>*Enterococcus spp*. 17.4%<br>*Streptococcus spp*. 23.9%<br>g– *E. coli* 7.3%<br>*enterobacteriaceae* 8.3%<br>*diphtheroids* 9.6%<br>f yeasts 8.7%<br>f molds 19.3%	Mobile phones	[65]
g+ *Staphylococcus spp.*<br>*S. epidermis* 31.1%<br>*S. aureus* 14.1%<br>*B. subtilis* 13.3%<br>*Micrococcus spp*. 14.1%<br>g– diphtheroids 6.7%		Mobile phones	[66]
g+ *Staphylococcus spp.*<br>CNS 25%<br>*S. aureus* 27%<br>*Bacillus spp*. 10%<br>*gram-positive bacilli*<br>(*none* spore forming) 7%<br>*gram-positive cocci* 13%<br>g– gram-negative bacilli 18%		Mobile phones	[67]
g+ *Staphylococcus spp*.<br>CNS 76.7%<br>*S. aureus* 11.9%<br>*Bacillus spp*. 33.2%<br>*Streptococcus spp*. 15.8%<br>g– *Pseudomonas spp*. 6.9%<br>*Acinetobacter spp*. 7.4 %<br>*Pantoae spp*. 6.9%		Mostly tablets	[68]
g+ *Staphylococcus spp*.<br>CNS 50%<br>MRSA 52.5%<br>MSSA 17.5 %<br>*Bacillus spp*. 42.5 %<br>*Micrococcus spp*. 10%<br>*Streptococcus viridans* 12.5%<br>g– *E. coli* 12.5%<br>*Klebsiella pneumoniae* 7.5%<br>diphtheroids 30%		Mobile phones	[46]
v rotavirus<br>v respiratory syncytial virus		Mobile phones	[69]
g+ *Staphylococcus spp.*<br>*S. epidermis* 46%<br>MSSA 22%		Mobile phones	[70]

Hospital	Community	Touchscreen device	Reference
g+ *Staphylococcus spp.*<br>CNS 81%<br>*Corynebacterium tuberculostearicum* 10%<br>g– enterobacteriaceae 7%<br>*gram*-negative oxidase *negative bacilli* 25%<br>*gram*-negative oxidase *positive bacilli* 31%<br>*sporulating bacilli* 24%<br>f fungi 6%		Mobile phones	[47]
g+ *Staphylococcus spp.*<br>*Micrococcus spp.*<br>*Enterococcus spp.*<br>*Kytococcus sedentarius*<br>g– *Clostridioides difficile*	g+ *Enterococcus aerogenes*<br>g– *E. coli*<br>Klebsiella *spp*.	Touchscreens	[48]
v respiratory syncytial virus 6%<br>v adenovirus 6%		Mobile phones	[71]
g+ *Staphylococcus spp.*<br>*S.* epidermis 26.7%<br>*Bacillus spp.* 13.3%<br>*Micrococcus spp*. 10%<br>*Enterococcus* spp. 6.7%<br>*non*-hem. *streptococcus* 6.7%<br>f non-albicans candida 16.7%		Mobile phones	[72]
g+ *Staphylococcus spp*.<br>CNS 73.5%<br>*Bacillus spp*. 16%<br>*Bacillus cereus* 7.5%<br>*Micrococcus luteus* 53%<br>*Corynebacterium spp*. 16%<br>Kocuria *spp*. 18.5%<br>*Paenibacillus lactis* 7.5%<br>g– *Acinetobacter lwoffu* 32.5%		Mobile phones	[49]
g+ *Staphylococcus spp*.<br>CNS 32.3%<br>*S. aureus* 18.1%<br>*Bacillus spp*. 13.4%<br>*Streptococcus viridans* 15.7%<br>*Corynebacterium spp*. 11.8%<br>g– *gram-negative bacilli* 6.3%		Mobile phones	[73]
g+ *Staphylococcus spp*.<br>CNS: 26.6%<br>*Bacillus spp*. 23.4%		Mobile phones	[74]
	g+: *Staphylococcus spp*.<br>CNS<br>*S.* aureus<br>g– *Enterobacter cloacae*<br>*Serratia spp*.	Different vending machines	[75]
g+ *Staphylococcus spp*.<br>CNS 11%<br>MRSA 19%<br>*Bacillus spp*. 5%<br>*Streptococcus viridans* 10%<br>g– *Pseudomonas aeruginosa* 9%<br>*E. coli* 17%<br>*Klebsiella pneumoniae* 9%<br>*Proteus spp*. 7%		Mobile phones	[76]

Hospital	Community	Touchscreen device	Reference
g+ *Staphylococcus spp*.<br>CNS 87.5%<br>*S. aureus* 5%<br>*Bacillus spp*. 60%<br>g– *Pseudomonas spp*. 50%<br>*Klebsiella spp*. 22.5%<br>*Acinetobacter spp*. 15%<br>*Proteus spp*. 12.5%		Mobile phones	[10]
g+ *Staphylococcus spp*.<br>CNS 64%<br>*S. aureus* 20%<br>*Bacillus spp*. 16%<br>*Micrococcus spp*. 8%<br>*Streptococcus spp*. 20%<br>g– diphtheroids 52%<br>f *Penicillium spp*. 8%		Mobile phones	[77]
	g– *coliforms* 30.9%<br>enterobacteriaceae 28.6%<br>f yeast and molds 53%	Mobile phones	[78
	g+ *Staphylococcus spp*. 75.3%<br>*Corynebacterium spp*. 6.2%<br>f yeast 16.4%	Mobile phones	[50]
g+ *S. aureus* 53.3%<br>*Bacillus spp*. 18.3%<br>g– *Proteus spp.*<br>*P. vulgaris* 36%<br>*P. mirabilis* 16%<br>*Salmonella paratyphi* A 12%<br>*Citrobacter freundii* 8%<br>gram-negative rods 23.3%		Mobile phones	[79]
g+ *Staphylococcus spp*.<br>CNS 22.9%<br>MSSA 17.0%<br>MRSA 8.1%<br>g– *Pseudomonas aerugin.* 7.6%<br>*E. coli* 22.9%<br>*Klebsiella aerogenes* 16.6%	g+ *Staphylococcus spp.*<br>CNS 28.2%<br>MSSA 18.7%<br>g– *Pseudomonas aerugin*.8.1%<br>*E. coli* 24.9%<br>*Klebsiella aerogenes* 16.3%	Mobile phones	[80]
g+ *Staphylococcus spp.*<br>*S. aureus* 32.2%<br>*Bacillus spp*. 43.5%<br>*Streptococcus spp*. 8%<br>g– *Pseudomonas spp*. 11.2%<br>f *Candida spp*. 6.4%<br>*Aspergillus niger* 11%		Mobile phones	[81]
	g+ *S. aureus* 56%	Mobile phones	[82]
g+ *Staphylococcus spp*<br>*S. epidermis* 42.2%<br>*S. saprophyticus* 11.1%<br>*Bacillus spp*. 57.8%<br>*Corynebacterium spp*. 6.7%	g+ *Staphylococcus spp.*<br>*S. epidermis* 66.7%<br>*S.* saprophyticus 11.1%<br>*Bacillus spp*. 40%<br>*Micrococcus spp*. 6.7%<br>*Corynebacterium spp*.22.2%	Mobile phones	[83]
g+ *Staphylococcus spp*.<br>CNS 76%<br>S. aureus 20%<br>*Bacillus spp*. 36%<br>*Micrococcus spp*. 36%<br>g– enteric bacteria 12%		Mobile phones	[13]

Hospital	Community	Touchscreen device	Reference
g+ *Bacillus spp.*<br>*Enterococcus faecalis*<br>g– *Pseudomonas spp*.<br>coliforms		Public touchscreens	[84]
g+ *S. aureus* 16.7%<br>*S.* epidermis 6.7%		Mobile phones	[85]
	g+ *Staphylococcus spp.*<br>*S. aureus* 44.8%<br>*S. epidermis* 27.6%<br>g– *E. coli* 20.7%	Mobile phones	[86]
g+ *Staphylococcus spp*.<br>CNS 22.6%<br>MSSA 6.7%<br>*Bacillus spp*. 11.5%<br>*Micrococcus spp*. 14.4%<br>g– *Pseudomonas spp*. 7.1%<br>*E. coli* 39.3%<br>*Klebsiella spp*. 17.9%		Mobile phones	[87]
g+ *Staphylococcus spp*.<br>CNS (*S. epidermis*) 28%<br>*S. aureus* 12%<br>*Bacillus spp*. 5%<br>g– *Pseudomonas aerugin.* 13%<br>*E. coli* 22%<br>*Klebsiella pneumoniae* 16%		Mobile phones	[88]
g+ *S. aureus* 45%	g+ *S. aureus* 25%	Mobile phones	[89]
	g+ *Staphylococcus spp.*<br>*S. epidermis* 100%<br>*S. aureus* 100%<br>*Bacillus spp*. 40%<br>g– *Pseudomonas spp*. 100%<br>*E. coli* 60%<br>f fungi	Cash dispenser	[90]
g+ *Staphylococcus spp.*<br>*S. aureus* 6%<br>g– *Pantoea spp*. 8.2%		Mobile phones and tablets	[17]
	g+ *Staphylococcus spp*.<br>CNS 47.9%<br>*S. aureus* 20.9%<br>*Bacillus spp*. 74.8%<br>g– *Pseudomonas aerugin.* 5%<br>*E. coli* 8%<br>*Klebsiella spp.*<br>*K. pneumoniae* 17.2%<br>*K. aerogenes* 16%	Mobile phones	[91]
	g+ *Staphylococcus spp*. 84.6%<br>*Bacillus spp*. 7.7%<br>*Enterococcus spp*. 7.7%	Mobile phones	[92]
g+ *Staphylococcus spp.*<br>*S. aureus* 34.6%<br>*S. epidermis* 23.1%<br>*S. saprophyticus* 15.4%<br>g– *Pseudomonas spp*. 20.5%		Mobile phones	[93]
g+ *Staphylococcus spp*. 85.2%<br>*S. epidermis* 72.2%<br>*S. capitis* 13.9%<br>*S. saprophyticus* 5.6%<br>*S. warneri* 5.6%<br>*S. xylosus* 5.6%<br>*Enterococcus spp*. 37%		Mobile phones	[51]

Hospital	Community	Touchscreen device	Reference
g+ *Staphylococcus spp*.<br>CNS 62%<br>*Bacillus spp*. 26%<br>*Micrococcus spp*. 41%<br>*Corynebacterium spp*. 5%<br>g– *Pseudomonas spp*. 10%<br>f fungi 6%		Mobile phones	[94]
g+ *Staphylococcus spp*.<br>CNS 62%<br>*S. aureus* 10%<br>*Bacillus cereus* 7%<br>*Enterococcus faecalis* 7%<br>g– *Acinetobacter spp*. 7%		Mobile phones	[18]
	g– *coliforms* 13.3%	Public touchscreens	[95]
	v SARS-CoV-2-RNA (25%)	Public touchscreen	[96]
	g+ *Staphylococcus spp*.<br>CNS 9%<br>*S. aureus* 21%,<br>*Bacillus spp*. 23%<br>g– *Pseudomonas aerugin.* 9%<br>*E. coli* 14%<br>*Klebsiella pneumoniae* 11%<br>*Proteus spp*. 8%	Cash dispenser	[52]
g+ *Staphylococcus spp*.<br>CNS 74.7%<br>f *Aspergillus niger* 13.7%<br>*Microsporum audounii* 5.6%		Mobile phones	[6]
g+ *Staphylococcus spp*.<br>CNS 19%<br>*Bacillus spp*. 39.5%<br>g– *Pseudomonas spp*. 5.5%<br>*E. coli* 15.5%<br>*Klebsiella spp*. 10.5%		Mobile phones	[97]
g+ *Staphylococcus spp*.<br>CNS 50%<br>*S. aureus* 24.5%<br>*Bacillus spp*. 14.3%		Mobile phones	[98]
g+ *Staphylococcus spp*.<br>CNS 25%<br>*Bacillus spp*. 15.5%		Mobile phones	[99]
	v SARS-CoV-2-RNA 19.1%	Cash dispenser	[100]
v SARS-CoV-2-RNA (25%)		Mostly tablets	[101]
	g+ *Staphylococcus aureus*<br>*Micrococcus spp*.<br>g– *Pseudomonas spp.*<br>*E. coli*<br>*Salmonella spp.*<br>*Serratia spp*.	Cash dispenser	[102]

Abbreviations:

g+: gram-positive bacteria

g–: gram-negative bacteria

f: fungi

v: virus

CNS: coagulase-negative Staphylococcus

MRSA: methicillin resistant *Staphylococcus aureus*

MSSA: methicillin-sensitive *Staphylococcus aureus*

VRE: Vancomycin-resistant Enterococcus

References

1. Brady RR, Verran J, Damani NN, Gibb AP. Review of mobile communication devices as potential reservoirs of nosocomial pathogens. J Hosp Infect. 2009 Apr;71(4):295-300. DOI: 10.1016/j.jhin.2008.12.009

2. Manning ML, Davis J, Sparnon E, Ballard RM. iPads, droids, and bugs: Infection prevention for mobile handheld devices at the point of care. Am J Infect Control. 2013 Nov;41(11):1073-6. DOI: 10.1016/j.ajic.2013.03.304

3. Hirsch EB, Raux BR, Lancaster JW, Mann RL, Leonard SN. Surface microbiology of the iPad tablet computer and the potential to serve as a fomite in both inpatient practice settings as well as outside of the hospital environment. PLoS One. 2014;9(10):e111250. DOI: 10.1371/journal.pone.0111250

4. Graveto JM, Costa PJ, Santos CI. Cell Phone Usage by Health Personnel – Preventive Strategies to Decrease Risk of Cross Infection in Clinical Context. Texto Contexto Enferm. 2018;25(1). DOI: 10.1590/0104-07072018005140016

5. Corrin T, Lin J, MacNaughton C, Mahato S, Rajendiran A. The role of mobile communication devices in the spread of infections within a clinical setting. Environ. Health Rev. 2016;59:63–70. DOI: 10.5864/d2016-014

6. Sadeeq T, Arikan A, Sanlidag T, Guler E, Suer K. Big Concern for Public Health: Microbial Contamination of Mobile Phones. J Infect Dev Ctries. 2021 Jun;15(6):798-804. DOI: 10.3855/jidc.13708

7. Omran A, Taha MS. Bacterial contamination of mobile phones among health care workers: A meta-analysis study. J Med Sci Res. 2020;2020:87–94.

8. Cremers-Pijpers S, van Rossum C, Dautzenberg M, Wertheim H, Tostmann A, Hopman J. Disinfecting handheld electronic devices with UV-C in a healthcare setting. Infect Prev Pract. 2021 Jun;3(2):100133. DOI: 10.1016/j.infpip.2021.100133

9. Beckstrom AC, Cleman PE, Cassis-Ghavami FL, Kamitsuka MD. Surveillance study of bacterial contamination of the parent's cell phone in the NICU and the effectiveness of an anti-microbial gel in reducing transmission to the hands. J Perinatol. 2013 Dec;33(12):960-3. DOI: 10.1038/jp.2013.108

10. Viveka VA. Isolation and Identification of Common Bacterial Contaminants in Mobile Phones Owned by Veterinary Undergraduate Students. J Heal, Medi Nur. 2017;35:92-105.

11. Jones M, Almeida G, Jones SL, Gibson KE. Prevalence and control of bacteria on single-user touchscreen mobile devices. Food Prot Trends. 2020;40:147-53.

12. Howell V, Thoppil A, Mariyaselvam M, Jones R, Young H, Sharma S, Blunt M, Young P. Disinfecting the iPad: evaluating effective methods. J Hosp Infect. 2014 Jun;87(2):77-83. DOI: 10.1016/j.jhin.2014.01.012

13. Koscova J, Hurnikova Z, Pistl J. Degree of Bacterial Contamination of Mobile Phone and Computer Keyboard Surfaces and Efficacy of Disinfection with Chlorhexidine Digluconate and Triclosan to Its Reduction. Int J Environ Res Public Health. 2018 Oct;15(10). DOI: 10.3390/ijerph15102238

14. Lieberman MT, Madden CM, Ma EJ, Fox JG. Evaluation of 6 Methods for Aerobic Bacterial Sanitization of Smartphones. J Am Assoc Lab Anim Sci. 2018;57:24–9.

15. Sriram S, Madan Kumar P, Swaminathan R, Venkatesh R, Menaka V. Effectiveness of isopropyl alcohol and ultraviolet-based sanitiser on decontamination of mobile phones used by dental personnel. J Patient Saf Infect Control. 2018;6:19. DOI: 10.4103/jpsic.jpsic_4_18

16. Resendiz M, Horseman TS, Lustik MB, Nahid A, West GF. Comparative effectiveness of rapid-cycle ultraviolet decontamination to chemical decontamination on high-touch communication devices. Am J Infect Control. 2019 Sep;47(9):1135-9. DOI: 10.1016/j.ajic.2019.02.022

17. Huffman S, Webb C, Spina SP. Investigation into the cleaning methods of smartphones and wearables from infectious contamination in a patient care environment (I-SWIPE). Am J Infect Control. 2020 May;48(5):545-9. DOI: 10.1016/j.ajic.2019.09.009

18. Malhotra S, Wlodarczyk J, Kuo C, Ngo C, Glucoft M, Sumulong I, Smit MA, Bender JM. Shining a light on the pathogenicity of health care providers' mobile phones: Use of a novel ultraviolet-C wave disinfection device. Am J Infect Control. 2020 Nov;48(11):1370-4. DOI: 10.1016/j.ajic.2020.05.040

19. Allen EM, McTague MF, Bay CP, Esposito JG, von Keudell A, Weaver MJ. The effectiveness of germicidal wipes and ultraviolet irradiation in reducing bacterial loads on electronic tablet devices used to obtain patient information in orthopaedic clinics: evaluation of tablet cleaning methods. J Hosp Infect. 2020 Jun;105(2):200-4. DOI: 10.1016/j.jhin.2020.04.014

20. Mathew JI, Cadnum JL, Sankar T, Jencson AL, Kundrapu S, Donskey CJ. Evaluation of an enclosed ultraviolet-C radiation device for decontamination of mobile handheld devices. Am J Infect Control. 2016 Jun;44(6):724-6. DOI: 10.1016/j.ajic.2015.12.043

21. Muniz de Oliveira R, da Rosa Gioppo NM, Oliveira de Carvalho J, Carvalho Oliveira F, Webster TJ, Marciano FR, Oliveira Lobo A. Decontamination of mobile phones and electronic devices for health care professionals using a chlorhexidine/carbomer 940® gel. Front Chem Sci Eng. 2019;13:192–8. DOI: 10.1007/s11705-018-1728-5

22. McGoldrick M. Preventing Contamination of Portable Computers. Home Healthc Now. 2016 Apr;34(4):221. DOI: 10.1097/NHH.0000000000000367

23. McGoldrick M. Preventing the Transfer of Pathogenic Organisms From the Use of Mobile Phones. Home Healthc Now. 2016 Jan;34(1):45. DOI: 10.1097/NHH.0000000000000330

24. Apple Inc. How to clean your Apple products. 2021 [Accessed 2021 Aug 5]. Available from: https://support.apple.com/en-us/HT204172

25. Samsung Electronics Co., Ltd. Keep your Galaxy device clean. 2021 [Accessed 2021 Aug 5]. Available from: https://www.samsung.com/us/support/answer/ANS00086342/

26. Fontana CR, Song X, Polymeri A, Goodson JM, Wang X, Soukos NS. The effect of blue light on periodontal biofilm growth in vitro. Lasers Med Sci. 2015 Nov;30(8):2077-86. DOI: 10.1007/s10103-015-1724-7

27. Alhmidi H, Cadnum JL, Piedrahita CT, John AR, Donskey CJ. Evaluation of an automated ultraviolet-C light disinfection device and patient hand hygiene for reduction of pathogen transfer from interactive touchscreen computer kiosks. Am J Infect Control. 2018 Apr;46(4):464-7. DOI: 10.1016/j.ajic.2017.09.032

28. Muzslay M, Yui S, Ali S, Wilson APR. Ultraviolet-C decontamination of hand-held tablet devices in the healthcare environment using the Codonics D6000™ disinfection system. J Hosp Infect. 2018 Nov;100(3):e60-e63. DOI: 10.1016/j.jhin.2018.04.002

29. The International Ultraviolet Association. Far UV-C Radiation: Current State-of Knowledge (White Paper). Bethesda, MD: IUVA; 2021 May 11. Available from: https://www.iuva.org/UV-C-Radiation-White-Paper

30. Hessling M, Haag R, Sieber N, Vatter P. The impact of far-UVC radiation (200–230 nm) on pathogens, cells, skin, and eyes – a collection and analysis of a hundred years of data. GMS Hyg Infect Control. 2021;16:Doc07. DOI: 10.3205/DGKH000378

31. Khazova M, Johnstone L, Naldzhiev D, O'Hagan JB. Survey of Home-Use UV Disinfection Products. Photochem Photobiol. 2021 May;97(3):560-5. DOI: 10.1111/php.13423

32. Stephenson CV, Moses BC, Wilcox WS. Ultraviolet irradiation of plastics. I. Degradation of physical properties. J Polym Sci. 1961;55:451–64. DOI: 10.1002/pol.1961.1205516204

33. Carrasco F, Pagès P, Pascual S, Colom X. Artificial aging of high-density polyethylene by ultraviolet irradiation. Euro Polymer J. 2001;37:1457–64. DOI: 10.1016/S0014-3057(00)00251-2

34. Gu H. Degradation of glass fibre/polyester composites after ultraviolet radiation. Materials & Design. 2008;29:1476–9. DOI: 10.1016/j.matdes.2007.07.010

35. Boubakri A, Guermazi N, Elleuch K, Ayedi HF. Study of UV-aging of thermoplastic polyurethane material. Mat Sci Eng: A. 2010;527:1649–54. DOI: 10.1016/j.msea.2010.01.014

36. Gomes de Castro Monsores K, Oliveira da Silva A, de Sant' Ana Oliveira S, Passos Rodrigues JG, Pondé Weber R. Influence of ultraviolet radiation on polymethylmethacrylate (PMMA). J Mat Res Tech. 2019;8:3713–8. DOI: 10.1016/j.jmrt.2019.06.023

37. Fischer HR, Semprimoschnig C, Mooney C, Rohr T, van Eck ERH, Verkuijlen MHW. Degradation mechanism of silicone glues under UV irradiation and options for designing materials with increased stability. Polym Degr Stab. 2013;98:720–6. DOI: 10.1016/j.polymdegradstab.2012.12.022

38. Heil H, Andress G, Schmechel R, Seggern H von, Steiger J, Bonrad K, Sprengard R. Sunlight stability of organic light-emitting diodes. J App Phy. 2005;97:124501. DOI: 10.1063/1.1935130

39. Askola J, Çalkin Y, Vaskuri A, Poikonen T, Ikonen E. Accelerated ageing of organic LED panels using ultraviolet exposure. Ligh Res Tech. 2019;51:1263–74. DOI: 10.1177/1477153518819654

40. Kwon SK, Baek JH, Choi HC, Kim SK, Lampande R, Pode R, Kwon JH. Degradation of OLED performance by exposure to UV irradiation. RSC Adv. 2019;9:42561–8. DOI: 10.1039/C9RA09730A

41. Ovca A, Rednak B, Godič Torkar K, Jevšnik M, Bauer M. Students' mobile phones – how clean are they? Sanitarno inženirstvo. 2012;6:6-18.

42. Albrecht UV, von Jan U, Sedlacek L, Groos S, Suerbaum S, Vonberg RP. Standardized, App-based disinfection of iPads in a clinical and nonclinical setting: comparative analysis. J Med Internet Res. 2013 Aug;15(8):e176. DOI: 10.2196/jmir.2643

43. Pal P, Roy A, Moore G, Muzslay M, Lee E, Alder S, Wilson P, Powles T, Wilson P, Kelly J. Keypad mobile phones are associated with a significant increased risk of microbial contamination compared to touchscreen phones. J Infect Prev. 2013;14:65-8. DOI: 10.1177/1757177413475903

44. Egert M, Späth K, Weik K, Kunzelmann H, Horn C, Kohl M, Blessing F. Bacteria on smartphone touchscreens in a German university setting and evaluation of two popular cleaning methods using commercially available cleaning products. Folia Microbiol (Praha). 2015 Mar;60(2):159-64. DOI: 10.1007/s12223-014-0350-2

45. Misgana G, Abdissa K, Abebe G. Bacterial contamination of mobile phones of health care workers at Jimma University Specialized Hospital, Jimma, South West Ethiopia. Int J Inf Cont. 2015;11:1–8.

46. Selim HS, Abaza AF. Microbial contamination of mobile phones in a health care setting in Alexandria, Egypt. GMS Hyg Infect Control. 2015;10:Doc03. DOI: 10.3205/dgkh000246

47. Murgier J, Coste JF, Cavaignac E, Bayle-Iniguez X, Chiron P, Bonnevialle P, Laffosse JM. Microbial flora on cell-phones in an orthopedic surgery room before and after decontamination. Orthop Traumatol Surg Res. 2016 Dec;102(8):1093-6. DOI: 10.1016/j.otsr.2016.09.014

48. Gerba CP, Wuollet AL, Raisanen P, Lopez GU. Bacterial contamination of computer touch screens. Am J Infect Control. 2016 Mar;44(3):358-60. DOI: 10.1016/j.ajic.2015.10.013

49. Kõljalg S, Mändar R, Sõber T, Rööp T, Mändar R. High level bacterial contamination of secondary school students' mobile phones. Germs. 2017 Jun;7(2):73-7. DOI: 10.18683/germs.2017.1111

50. Di Lodovico S, Del Vecchio A, Cataldi V, Di Campli E, Di Bartolomeo S, Cellini L, Di Giulio M. Microbial Contamination of Smartphone Touchscreens of Italian University Students. Curr Microbiol. 2018 Mar;75(3):336-42. DOI: 10.1007/s00284-017-1385-9

51. Cicciarella Modica D, Maurici M, D'Alò GL, Mozzetti C, Messina A, Distefano A, Pica F, De Filippis P. Taking Screenshots of the Invisible: A Study on Bacterial Contamination of Mobile Phones from University Students of Healthcare Professions in Rome, Italy. Microorganisms. 2020 Jul;8(7):1075. DOI: 10.3390/microorganisms8071075

52. Osarenmwinda O, Ofure Blessing O. Quantification, Variability Assessment of Bacterial Pollution and Public Health Hazards Linked to Users of Automated Teller Machines in Ekpoma, Edo State-Nigeria. IJMB. 2020;5:34-40. DOI: 10.11648/j.ijmb.20200501.16

53. Ulger F, Dilek A, Esen S, Sunbul M, Leblebicioglu H. Are healthcare workers' mobile phones a potential source of nosocomial infections? Review of the literature. J Infect Dev Ctries. 2015 Oct;9(10):1046-53. DOI: 10.3855/jidc.6104

54. Olsen M, Campos M, Lohning A, Jones P, Legget J, Bannach-Brown A, McKirdy S, Alghafri R, Tajouri L. Mobile phones represent a pathway for microbial transmission: A scoping review. Travel Med Infect Dis. 2020 May-Jun;35:101704. DOI: 10.1016/j.tmaid.2020.101704

55. Mukhtar-Yola M, Andrew B. Are mobile phones of health care workers portals of pathogenic organisms causing hospital acquired infections in intensive care units? A mini systematic review. Nig. J. Paed. 2020;47:207-14. DOI: 10.4314/njp.v47i3.3

56. Boucher HW, Talbot GH, Bradley JS, Edwards JE, Gilbert D, Rice LB, Scheld M, Spellberg B, Bartlett J. Bad bugs, no drugs: no ESKAPE! An update from the Infectious Diseases Society of America. Clin Infect Dis. 2009 Jan;48(1):1-12. DOI: 10.1086/595011

57. DIN EN 14897:2007-09 – Water conditioning equipment inside buildings – Devices using mercury low-pressure ultraviolet radiators – Requirements for performance, safety and testing. Berlin: Beuth; 2007.

58. Akinyemi KO, Atapu AD, Adetona OO, Coker AO. The potential role of mobile phones in the spread of bacterial infections. J Infect Dev Ctries. 2009 Sep;3(8):628-32. DOI: 10.3855/jidc.556

59. Brady RR, Hunt AC, Visvanathan A, Rodrigues MA, Graham C, Rae C, Kalima P, Paterson HM, Gibb AP. Mobile phone technology and hospitalized patients: a cross-sectional surveillance study of bacterial colonization, and patient opinions and behaviours. Clin Microbiol Infect. 2011 Jun;17(6):830-5. DOI: 10.1111/j.1469-0691.2011.03493.x

60. Kiedrowski LM, Perisetti A, Loock MH, Khaitsa ML, Guerrero DM. Disinfection of iPad to reduce contamination with Clostridium difficile and methicillin-resistant Staphylococcus aureus. Am J Infect Control. 2013 Nov;41(11):1136-7. DOI: 10.1016/j.ajic.2013.01.030

61. La Fauci V. The Possible Role of Mobile Phones in Spreading Microorganisms in Hospitals. J Microb Biochem Technol. 2014;6(6):334-6. DOI: 10.4172/1948-5948.1000164

62. Meadow JF, Altrichter AE, Green JL. Mobile phones carry the personal microbiome of their owners. PeerJ. 2014;2:e447. DOI: 10.7717/peerj.447

63. Mark D, Leonard C, Breen H, Graydon R, O'Gorman C, Kirk S. Mobile phones in clinical practice: reducing the risk of bacterial contamination. Int J Clin Pract. 2014 Sep;68(9):1060-4. DOI: 10.1111/ijcp.12448

64. Danen P, Duarte A, Barroso H. Comparison of microflora present in mobile phones and in the hands of its owners: detection of antibiotic resistant bacteria. In: International Meeting in Forensic Sciences; 2014 May; Almada, Portugal. Available from: https://www.researchgate.net/publication/263044335_International_Meeting_in_Forensic_Sciences_C_Comparison_of_microflora_present_in_mobile_phones_and_in_the_hands_of_its_owners_detection_of_antibiotic_resistant_bacteria

65. Koroglu M, Gunal S, Yildiz F, Savas M, Ozer A, Altindis M. Comparison of keypads and touch-screen mobile phones/devices as potential risk for microbial contamination. J Infect Dev Ctries. 2015 Dec;9(12):1308-14. DOI: 10.3855/jidc.6171

66. Pal K, Chatterjee M, Sen P, Adhya S. Cell Phones of Health Care Professionals: A Silent Source of Bacteria. Nat J Lab Med. 2015;4:33-8.

67. Elmanama A, Hassona I, Marouf A. Microbial Load of Touchscreen Mobile Phones Used by University Students and Healthcare Staff. JAAU. 2015;1:1–18. DOI: 10.12816/0020268

68. Khan A, Rao A, Reyes-Sacin C, Hayakawa K, Szpunar S, Riederer K, Kaye K, Fishbain JT, Levine D. Use of portable electronic devices in a hospital setting and their potential for bacterial colonization. Am J Infect Control. 2015 Mar;43(3):286-8. DOI: 10.1016/j.ajic.2014.11.013

69. Pillet S, Berthelot P, Gagneux-Brunon A, Mory O, Gay C, Viallon A, Lucht F, Pozzetto B, Botelho-Nevers E. Contamination of healthcare workers' mobile phones by epidemic viruses. Clin Microbiol Infect. 2016 May;22(5):456.e1-6. DOI: 10.1016/j.cmi.2015.12.008

70. Thomas W, Oller A. Staphylococcus and Pseudomonas Isolated from Mobile Phones and Cheek and Ear Locales. BJMMR. 2016;11:1-8. DOI: 10.9734/BJMMR/2016/20616

71. Cavari Y, Kaplan O, Zander A, Hazan G, Shemer-Avni Y, Borer A. Healthcare workers mobile phone usage: A potential risk for viral contamination. Surveillance pilot study. Infect Dis (Lond). 2016;48(6):432-5. DOI: 10.3109/23744235.2015.1133926

72. Jalalmanesh S, Darvishi M, Rahimi M, Akhlaghdoust M. Contamination of Senior Medical Students' Cell Phones by Nosocomial Infections – A Survey in a University-Affiliated Hospital in Tehran. Shiraz E-Med J. 2017. DOI: 10.5812/semj.43920

73. Mohd R, Zaman Q, Refat N, Helmi M. Isolation of bacteria from mobile phones before and after decontamination: Study carried out at King Abdulaziz University, Jeddah, Saudi Arabia. Afr J Microbiol Res. 2017;11:1371-8.

74. Raza I, Raza A, Razaa SA, Sadar AB, Qureshi AU, Talib U, Chi G. Surface Microbiology of Smartphone Screen Protectors Among Healthcare Professionals. Cureus. 2017;9:e1989. DOI: 10.7759/cureus.1989

75. Dakroub R, Nawas T. Vending machine buttons and touchscreens: A surface colonized by pathogenic bacteria. Int J Inn Appl Res. 2017;5:82-8.

76. AL-Safaar MA. Prevalence of Methicilin-Resistant Staphylococcus aureus (MRSA) in Mobile Phone of Healthcare Workers in Baghdad Teaching Hospital. J Med Sci Cli Res. 2017;05:17796-803. DOI: 10.18535/jmscr/v5i2.98

77. Canales MB, Craig GC, Boyd Jr J, Markovic M, Chmielewski RA. Dissemination of Pathogens by Mobile Phones in a Single Hospital. ReconRev. 2017. DOI: 10.15438/rr.7.3.192

78. Martínez-Gonzáles N, Solorzano-Ibarra F, Cabrera-Díaz E, Gutiérrez-González P, Martínez-Chávez L, Pérez-Montaño J, Martínez-Cárdenasa C. Microbial contamination on cell phones used by undergraduate students. Cana J Inf Cont. 2018;32:211-6.

79. Almugadam BS, Ahmed HM, Osman MB, Omer S. Frequency of MRSA Isolates in Mobile Phones, Ears and Hands of Healthcare Workers. J Antimicrob Agents. 2018. DOI: 10.4172/2472-1212.1000161

80. Ghatole KP. Mobile Phones – Do We Need Decontamination? jebmh. 2018;5:425-8. DOI: 10.18410/jebmh/2018/86

81. Tamilpavai R, Rajendra Prasad B, Uma C, Sivagurunathan P, Muthulakshmi K. Incidence Of Nosocomial Pathogens On The Mobile Phones. Indo Am J Phar Sci. 2018;5:272-7. DOI: 10.5281/zenodo.1225106

82. Khadka S, Adhikari S, Sapkota S, Shrestha P. Methicillin-Resistant Staphylococcus aureus Associated with Mobile Phones. SOJMID. 2018;6:1-6. DOI: 10.15226/sojmid/6/1/00190

83. Movahhed T, Dehghani M, Ghoddusi T. Evaluation of microbial contamination of mobile phones and computer mice and keyboards in a dental school. J Dental Mat Tech. 2018;7:78-82.

84. Smith A, Matewele P. Poo found on every McDonald’s touchscreen tested. 2018 [Accessed 2021 Aug 5]. Aviable from: https://metro.co.uk/2018/11/28/poo-found-on-every-mcdonalds-touchscreen-tested-8178486/

85. Mohammed TK, Jwad MA, Kamal O, Abbas AH, Alabbas AS. Isolation of Some Pathogenic Bacteria and Fungi From Student′s Mobile Phones (Part I). Indian J Public Health Res Dev. 2019;10(10):2108.

86. Kawakib I. Bacterial contamination on mobile phone devices of undergraduate students in Al-Qurna Education College – Basrah University. World J Phar Res. 2019;8:1-12.

87. Hadi OM, Fadel RH, Sayal RA, Khudhair SH. The role of mobile phones in the transmission of Methicillin-Resistant Staphylococcus aureus (MRSA) among the students and staff of the College of Health and Medical Technology/ Kufa in Najaf, Iraq. J Glob Phar Tech. 2019;11:82-7.

88. Taher N. Pathogenic Bacteria Isolated from Personal Cell Phones of Health Care Staff in Iraqi Hospitals. J Pure Appl Microbiol. 2019;13:1145-50. DOI: 10.22207/JPAM.13.2.53

89. Chimbekujwo KI, Bashir M, Aishat M, Bilyaminu M. Prevalence of Methicillin Resistant Staphylococcus aureus (MRSA) Associated with Mobile Phone. Inter Res J Advan Engi Sci. 2019;4:270-2.

90. Acharjee M, Akter T, Tabassum N, Rahaman MM, Noor R. Prevalence of Methicillin and Vancomycin resistant Staphylococcus aureus on the touchscreen of automated teller machines in Dhaka city. Bangla. J. Microbiol. 2019;36:23-7. DOI: 10.3329/bjm.v36i1.44279

91. Hikmah N’, Anuar TS. Mobile Phones: A Possible Vehicle of Bacterial Transmission in a Higher Learning Institution in Malaysia. Malays J Med Sci. 2020 Mar;27(2):151-8. DOI: 10.21315/mjms2020.27.2.15

92. Campista-León S, Garcia-Guerrero JT, Olimón-Andalón V, Peinado-Guevara LI. Isolation of Gram-Positive, Antibiotic-Resistant Bacteria from Tactile Mobile Phones in a Northwestern Mexican City. J Community Health. 2020 Oct;45(5):1050-60. DOI: 10.1007/s10900-020-00829-5

93. Edrees WH, Sadeq Al-Awar M. Bacterial contamination of mobile phones of medical laboratory workers at Sana'a city, Yemen and their antimicrobial susceptibility. J Phar Pharmaco Res. 2020;8:591-9.

94. Qureshi NQ, Mufarrih SH, Irfan S, Rashid RH, Zubairi AJ, Sadruddin A, Ahmed I, Noordin S. Mobile phones in the orthopedic operating room: Microbial colonization and antimicrobial resistance. World J Orthop. 2020 May;11(5):252-64. DOI: 10.5312/wjo.v11.i5.252

95. Lam D, Moos M, Meldrum R. Surface microbiology of the electronic menu in all-you-can-eat sushi restaurants in Toronto, Ontario. Environ. Health Rev. 2020;63:14-20. DOI: 10.5864/d2020-003

96. Gholipour S, Nikaeen M, Mohammadi Manesh R, Aboutalebian S, Shamsizadeh Z, Nasri E, Mirhendi H. Severe Acute Respiratory Syndrome Coronavirus 2 (SARS-CoV-2) Contamination of High-touch Surfaces in Field Settings. Biomed Environ Sci. 2020;33:925-9. DOI: 10.3967/bes2020.126

97. Shukla P, Khalid B, Yaqoob S, Ahmad S. Involvement of Mobile phones as a source for Nosocomial infections. Asian J Med Sci. 2021;12:126-9. DOI: 10.3126/ajms.v12i7.34788

98. Mushabati NA, Samutela MT, Yamba K, Ngulube J, Nakazwe R, Nkhoma P, Kalonda A. Bacterial contamination of mobile phones of healthcare workers at the University Teaching Hospital, Lusaka, Zambia. Infect Prev Pract. 2021 Jun;3(2):100126. DOI: 10.1016/j.infpip.2021.100126

99. Kuriyama A, Fujii H, Hotta A, Asanuma R, Irie H. Prevalence of bacterial contamination of touchscreens and posterior surfaces of smartphones owned by healthcare workers: a cross-sectional study. BMC Infect Dis. 2021 Jul;21(1):681. DOI: 10.1186/s12879-021-06379-y

100. da Silva SJR, do Nascimento JCF, dos Santos Reis WPM, da Silva CTA, da Silva PG, Germano Mendes RP, Mendonça AA, Rodrigues Santos BN, de Magalhães JJF, Kohl A, Pena L. Widespread Contamination of SARS-CoV-2 on Highly Touched Surfaces in Brazil During the Second Wave of the COVID-19 Pandemic [Preprint]. medRxiv. 2021 Jan 01. DOI: 10.1101/2021.06.14.21258894

101. Nelson A, Kassimatis J, Estoque J, Yang C, McKee G, Bryce E, Hoang L, Daly P, Lysyshyn M, Hayden AS, Harding J, Boraston S, Dawar M, Schwandt M. Environmental detection of severe acute respiratory syndrome coronavirus 2 (SARS-CoV-2) from medical equipment in long-term care facilities undergoing COVID-19 outbreaks. Am J Infect Control. 2021 Feb;49(2):265-8. DOI: 10.1016/j.ajic.2020.07.001

102. Dawodu OG, Akanbi RB. Isolation and identification of microorganisms associated with automated teller machines on Federal Polytechnic Ede campus. PLoS One. 2021;16(8):e0254658. DOI: 10.1371/journal.pone.0254658

103. Kowalski W. Ultraviolet Germicidal Irradiation Handbook. Berlin, Heidelberg: Springer; 2009.

104. Santos AL, Oliveira V, Baptista I, Henriques I, Gomes NC, Almeida A, Correia A, Cunha Â. Wavelength dependence of biological damage induced by UV radiation on bacteria. Arch Microbiol. 2013 Jan;195(1):63-74. DOI: 10.1007/s00203-012-0847-5

105. Kelland LR, Moss SH, Davies DJ. An action spectrum for ultraviolet radiation-induced membrane damage in Escherichia coli K-12. Photochem Photobiol. 1983 Mar;37(3):301-6. DOI: 10.1111/j.1751-1097.1983.tb04477.x

106. Yagi N, Mori M, Hamamoto A, Nakano M, Akutagawa M, Tachibana S, Takahashi A, Ikehara T, Kinouchi Y. Sterilization using 365 nm UV-LED. Annu Int Conf IEEE Eng Med Biol Soc. 2007;2007:5842-5. DOI: 10.1109/IEMBS.2007.4353676

107. Lui GY, Roser D, Corkish R, Ashbolt NJ, Stuetz R. Point-of-use water disinfection using ultraviolet and visible light-emitting diodes. Sci Total Environ. 2016 May;553:626-35. DOI: 10.1016/j.scitotenv.2016.02.039

108. Takada A, Matsushita K, Horioka S, Furuichi Y, Sumi Y. Bactericidal effects of 310 nm ultraviolet light-emitting diode irradiation on oral bacteria. BMC Oral Health. 2017 Jun;17(1):96. DOI: 10.1186/s12903-017-0382-5

109. Matafonova GG, Batoev VB, Astakhova SA, Gómez M, Christofi N. Efficiency of KrCl excilamp (222 nm) for inactivation of bacteria in suspension. Lett Appl Microbiol. 2008 Dec;47(6):508-13. DOI: 10.1111/j.1472-765X.2008.02461.x
